# Application of Ecological Network Theory to the Human Microbiome

**DOI:** 10.1155/2008/839501

**Published:** 2008-10-29

**Authors:** James A. Foster, Stephen M. Krone, Larry J. Forney

**Affiliations:** ^1^Department of Biological Sciences, University of Idaho, Moscow, ID 83844-3051, USA; ^2^The Initiative for Bioinformatics and Evolutionary STudies (IBEST), University of Idaho, Moscow, ID 83844-3051, USA; ^3^Department of Mathematics, University of Idaho, Moscow, ID 83844-3051, USA

## Abstract

In healthy humans, many microbial consortia constitute rich ecosystems with dozens to hundreds of species, finely tuned to functions relevant to human health. Medical interventions, lifestyle changes, and the normal rhythms of life sometimes upset the balance in microbial ecosystems, facilitating pathogen invasions or causing other clinically relevant problems. Some diseases, such as bacterial vaginosis, have exactly this sort of community etiology. Mathematical network theory is ideal for studying the ecological networks of interacting species that comprise the human microbiome. Theoretical networks require little consortia specific data to provide insight into both normal and disturbed microbial community functions, but it is easy to incorporate additional empirical data as it becomes available. We argue that understanding some diseases, such as bacterial vaginosis, requires a shift of focus from individual bacteria to (mathematical) networks of interacting populations, and that known emergent properties of these networks will provide insights that would be otherwise elusive.

## 1. INTRODUCTION

The microbiota normally associated with the human body have an important influence on human development, physiology, immunity, and nutrition [1–6]. Also, communities of commensal and mutualistic bacteria associated with the human body constitute the first line of defense against infection by competitively excluding invasive nonindigenous organisms that cause disease. Yet despite their importance, surprisingly little is known about the composition of resident communities, how they differ between individual hosts or host environments, or such ecological relationships of constituent members as trophic interdependencies. Even so, human associated communities are likely to resemble those found in other habitats in at least four fundamentally important ways. First, natural microbial communities tend to be diverse in terms of species composition and physiological potential. Second, the flow of energy and nutrients through the system follows basic principles of microbial physiology, which results in the existence of trophic webs. Third, nutritional interdependencies exist wherein the “cross-feeding” of various vitamins, amino acids, and other cofactors occurs. And fourth, all ecological niches are occupied resulting in a relatively stable community composition. Armed with this information one can begin to postulate how external forces (e.g., invasive species such as nonindigenous microorganisms and pathogens) or treatments (e.g., the administration of antibiotics or changes in host diet) might affect the species composition and function of microbial communities that constitute the human microbiome.

Microbial communities can be viewed as mathematical networks with structural features that reflect how the networks developed and predict their responses to perturbations. In this paper, we will introduce the basic mathematical foundations of networks and briefly summarize some of their important structural properties. This approach to understanding microbial communities of the human microbiome is admittedly speculative, largely because of the lack of knowledge about community composition and species interactions in the human microbiome. Even so, it is based on a growing body of research on evolving networks and may constitute a useful conceptual framework for understanding how these communities help maintain human health and how disturbances of the community structure and function could increase susceptibility to infectious disease. To illustrate the importance of ecological networks in the human microbiome, we will describe the biology of microbiota of the human vagina and how disturbances to these communities may account for the clinical syndrome known as bacterial vaginosis.

## 2. MUTUALISTIC RELATIONSHIPS OF THE VAGINAL MICROBIOME

The human vagina and the bacterial communities that reside therein, form a finely balanced mutualistic association. Previous studies indicate that indigenous bacterial populations play a key role in preventing colonization by “undesirable” organisms, including those responsible for bacterial vaginosis, yeast infections, sexually transmitted diseases, and urinary tract infections [7–12]. Historically, lactobacilli have been thought to be the keystone species of vaginal communities in reproductive-age women, both in the sense of being the dominant species and in the sense of being the species with the greatest impact on the vaginal ecosystem. These microorganisms benefit the host by producing lactic acid as a fermentation product that accumulates in the environment and lowers the pH to ~4.5 [13]. While a wide range of other species are known to be members of vaginal bacterial communities, their ecological functions are largely unknown, as is the total number of species present. The host provides benefit to the microbial communities by providing all the nutrients needed to support bacterial growth. This is of obvious importance since bacteria are continually shed from the body in vaginal secretions, and bacterial growth must occur to replenish their numbers. Some of the required nutrients are derived from sloughed cells, while others are from glandular secretions. Surprisingly, the precise composition and the concentrations of various constituents are poorly understood, and this is an important knowledge gap. Nonetheless, the data available indicate that there are proteins and carbohydrates of various kinds in vaginal secretions, as well as urea, K+, Na+, and, Cl− [14] and it seems likely that various amino acids, peptides, and monosaccharides are also present. The symbiotic relationships between host and bacterial populations seem likely to be mutualisms, with each species benefiting from the presence of the other. (It should be noted that bacterial populations of the human microbiome are often referred to as commensal bacteria, which implies that only one member of the association benefits while the other is unaffected. In many cases, if not all, this is probably an incorrect characterization of the ecological relationship between the two members.)

## 3. ETIOLOGY OF BACTERIAL VAGINOSIS: A DISEASE LINKED TO COMMUNITY DISTURBANCES

Bacterial vaginosis (BV) is a syndrome that is often characterized as a disturbed microbial community [15] although it is most often diagnosed based on the occurrence of three of the following four criteria: (a) homogeneous, white adherent vaginal discharge; (b) a vaginal pH > 4.5; (c) detection of “clue cells” by microscopy; and (d) the presence of an amine odor upon addition of KOH to vaginal secretions [16]. Intensive efforts to identify etiological agents have thus far been unsuccessful, and it has been suggested that the disturbed communities themselves may account for the observed symptoms.

BV has important consequences for women's health. The prevalence of BV among reproductive-age women ranges from 29% in U.S. population-based surveys to over 50% in rural Ugandan villages [17]. It has been associated with an increased risk of preterm delivery, first trimester miscarriage in women undergoing in vitro fertilization, chorioamnionitis, endometritis, and pelvic inflammatory disease (PID) [18–23]. Moreover, BV increases the risk of acquiring *Neisseria gonorrhoeae* and other sexually transmitted diseases [11, 24] including HIV [8, 25].

Historically, BV has been associated with depleted numbers of *Lactobacillus* spp. and an elevated vaginal pH [26, 27]. However, this simple view has been challenged [28] by recent findings that showed that the vaginal communities of many normal and healthy Caucasian, black, and Japanese women lack appreciable numbers of *Lactobacillus* spp., but instead include other taxa of lactic acid producing bacteria (LAB) [29, 30]. This has two important implications. First, an important ecological benefit to the host—maintenance of a low vaginal pH—is conserved among individual women, although the species composition of the microbial communities can vary. This is consistent with the consensus viewpoint that a low pH environment in the vagina is a key mechanism for defending the host against potential pathogens. And second, factors that alter the species composition, the physiological activities of bacterial populations, or the overall community function (reducing the pH of the local environment), could lead to the symptoms associated with BV.

Previous studies have established that several distinct kinds of vaginal communities occur in Caucasian and black women in North America [29, 30], and Japanese women in Tokyo, Japan [Zhou, 2008; unpublished]. Since vaginal bacterial communities differ in species composition [30–33], they are likely to differ in how they respond to disturbances, and disruptions of ecological equilibria may increase risk to invasion by infectious agents. Conceptually this is important since vaginal communities continually experience various kinds of chronic and acute disturbances such as the use of antibiotics and hormonal contraceptives, sexual intercourse, douching, menstruation, and many others.

## 4. NETWORK APPROACHES TO UNDERSTANDING THE HUMAN VAGINAL MICROBIOME

**Figure 1:**
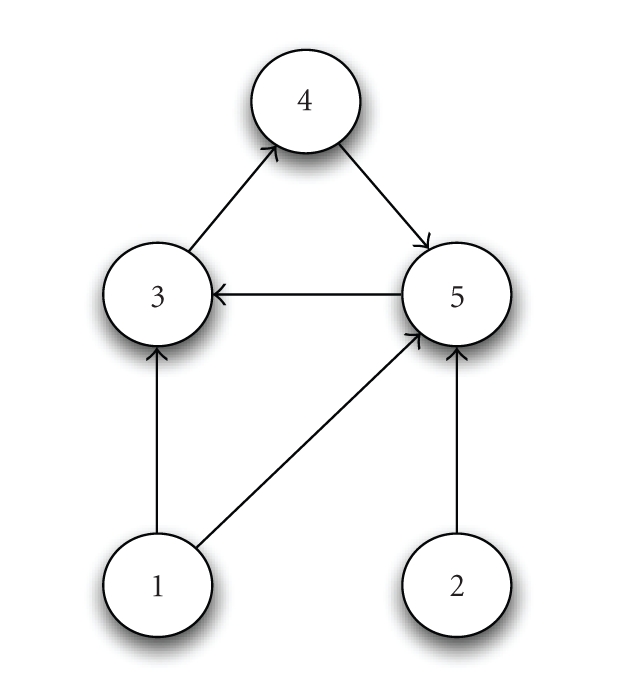
A hypothetical trophic web with five species. Species 1 and 2 are “grazers” at the bottom level, which acquire nutrients directly from the environment and provide nutrients to species 3 and 5. Species 3, 4, and 5 form a dependent cycle, with 3 and 5 at the second level of the web and 4 at the final level.

By analogy with microbial communities in other ecosystems, we postulate that a complex food web exists among member species of vaginal bacterial communities, and that various populations occur in distinct trophic levels. Given that the resource pool is diverse (as described above), it is reasonable to project that the species composition, expressed physiological traits, and kinds of nutritional interdependencies of vaginal bacterial populations are strongly influenced by the kinds of nutrients available in the vagina. This implies that host characteristics could be an important “driver” of microbial ecosystems, while the members of the microbial community are stratified in such a way that one or more populations are primary consumers, while others consume their metabolites, and so on. The result is a “network” that reflects the flow of energy and nutrients through the ecosystem in which the configuration and strengths of ecological interactions determine the stability and resilience of the community. Such networks are commonly referred to as microbial trophic webs (Figure 1).

In dissimilatory microbial trophic webs a few species specialize in breaking down larger, more complex organic molecules into smaller molecules [34, page 102]. These specialists may require little assistance from other species. There are likely to be more pathways (and microbial species) able to metabolize these smaller molecules, and still other species to consume the resulting metabolites. If complete mineralization of carbon sources occurs, then carbon dioxide is produced, but in the absence of suitable terminal electron acceptors, fermentation products (such as lactic acid) accumulate in the environment.

Some populations in dissimilatory consortia may have secondary roles that regulate the growth and function of other populations in the consortia. For example, one population may produce growth factors such as amino acids, peptides, or vitamins that are used, and sometimes required, for other populations to grow. Indeed, lactobacilli are notoriously fastidious and have complex nutritional requirements [35–37]. This sort of nutritional cross-feeding represents a “positive feedback loop.” In contrast, various small molecules that disrupt membrane function, antibiotics, and bacteriocidal proteins [38] constitute “negative feedback loops.” These positive and negative feedback loops play a role in governing the size of different bacterial populations and their activities. To understand such a complex network, one may very well have to adopt a systems approach such as that described below [39].

Since there may be very few specialist species at the base of microbial trophic webs, assembly rules may be strongly influenced by priority effects. A priority effect [40, page 247] is the influence that one species exerts on whether another can endure in an environment, simply by being there first. Assembly rules describe the order in which species tend to occupy habitats. For example, the first species to colonize a microbial ecosystem that specializes in catabolizing the dominant nutrient or nutrients may determine which new nutrients are then available, and thereby constrain which other species can successfully colonize the habitat and persist.

## 5. MATHEMATICAL REPRESENTATION OF NETWORKS

Microbial trophic webs of the human microbiome are instances of a more general abstract structure: mathematical networks. In ecology, trophic webs are typically visualized as nodes on a graph representing individual species that are connected by directed edges that indicate who is dependent on whom for nutrition. These webs are sometimes called “food webs,” with a tacit assumption that the relationship is one of who eats whom. Predatory-prey relationships exist at all scales of life. But both macro- and microbial trophic relationships are much richer than predation alone. For example, species interactions often involve cross-feeding, where each species acquires nutrients, or compounds that inhibit growth, that are produced by other species. In microbial systems, these indirect products are molecular, while in macrobial systems they may be much larger.

Collections of nodes and edges such as those used to visualize trophic webs are instances of mathematical networks. One useful characteristic of this mathematical abstraction is its general applicability. Any collection of “individuals” and “relationships” can be expressed and analyzed as a network, regardless of details about the individuals or the relations. In particular, networks are not limited to trophic webs.

**Figure 2:**
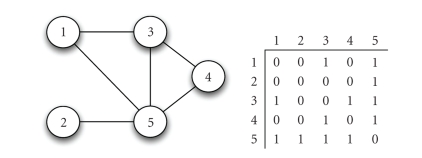
Mathematical network with undirected edges, representing the structure of the trophic web in Figure 1, pictorially and as an equivalent adjacency matrix. The connectivity of the nodes is one for node 2, two for nodes 1 and 4, three for node 3, and four for node 5 (which is a hub node).

**Figure 3:**
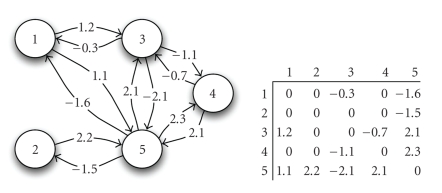
Directed graph representing (hypothetical) strengths of species interactions and the corresponding matrix of interaction strengths. Positive (negative) values indicate increase (decrease) in receiving species' fitness. Units of interaction are unspecified in this example, but may be observed changes in biomass. For example, species 1 may produce a metabolite beneficial to species 3(*a*_31_ = 1.2), while 3 occasionally harms 1(*a*_13_ = −0.3) while consuming the metabolite. Species 3 and 4 are competitors, 5 and 4 are mutualists, and other pairs resemble predator/prey.

The simplest mathematical networks indicate only whether or not two nodes are connected by an edge by setting the corresponding “adjacency” term to 1 or 0; thus, *a*_*i*,*j*_ is set to 1 if the *i*th individual is connected to the *j*th individual, and to 0 if they are not connected. These networks are often summarized in an adjacency matrix A with the term *a*_*i*,*j*_ appearing in the *i*th row and *j*th column. These connections are undirected when the matrix is symmetric, meaning that *a*_*i*,*j*_ = *a*_*j*,*i*_ (visually, reflecting the matrix across the main diagonal leaves it unchanged). One can represent additional information about the relation between two individuals by letting the matrix entries be numbers other than 0 and 1 (Figure 2). For example, an ecological network could correspond to a system of Lotka-Volterra differential equations describing species interactions *d* *u*_*i*_/*d* *t* = *u*_*i*_(*r*_*i*_ + ∑_*j*_ *a*_*i*,*j*_ *u*_*j*_) where *r*_*i*_ is the intrinsic growth rate of species *i* and *a*_*i*,*j*_ is the “effect” of species *j* on species *i*. Here, the interactions are described by a matrix A = (*a*_*i*,*j*_) of real numbers. For example, if *i* is a prey species and *j* a predator, we would have *a*_*i*,*j*_ < 0 and *a*_*j*,*i*_ > 0 (Figure 3). Food webs are special cases of ecological networks in which the interactions are all of predator-prey type with predators in one trophic level feeding on prey from a lower level (Figure 1).

It can extremely difficult to obtain information about trophic interactions (especially interaction strengths) in real ecological networks. However, it is becoming easier to gather quantitative data for networks given advances in high throughput sequencing technologies and sophisticated computational biology algorithms. For example, as more annotated genomes become available, it becomes easier to form hypotheses about potential metabolic pathways. It is encouraging that genome annotation, comparative genomics, and hypothetical pathway reconstruction are autocatalytic, each improving the accuracy and efficiency of the others. With such positive feedback, we anticipate that it will become increasingly easy to parameterize network models accurately.

Surprisingly, however, one does not need accurate parameters in an abstract network, since the network structure alone can tell one a great deal about the system that it represents. A characteristic that matters in all networks is the number of links or “connectedness” of each node, and this of course varies from one node to another within a network [41]. For example, a property of many natural networks is that they are “scale free,” roughly meaning that there is no single degree of connectedness that is characteristic of the network. In scale free networks, most nodes are connected to a small number of other nodes, and a small number of nodes act as “hubs” in that they are connected to many nodes. A scale free network is usually robust to the removal of randomly selected nodes but can be violently destabilized when hub nodes are removed. In a very real way, these hubs are analogous to keystone species in biological ecosystems. When the population size or activity of a keystone species is changed, or the species is entirely removed, dramatic changes occur in the varieties and population densities of all other species in the community.

It is even possible to learn a great deal with neither accurate graph topologies nor extensive empirical parameterization. Theoreticians construct artificial networks with different types of assembly rules, essentially reverse engineering the abstractions of natural networks. This discipline has been aptly termed the statistical mechanics of complex networks [42].

Remarkably, two informative properties consistently emerge from such simulations. First, in both real and simulated ecological networks one finds a “many weak, few strong” pattern in which most, but not all, species interactions are weak. Specifically, the average interaction strength (average of |*a*_*i*,*j*_| 's) times the square root of the average number of edges per node, often converges to a constant over time [43–45]. A second “emergent” property is that networks tend to evolve to the point where they are at the brink of instability, being in some sense most productive when living on the edge. Extinction events in an ecological network, either by “natural” means or by artificially removing nodes, typically lead to occasional avalanches of secondary extinctions [43, 46]. In fact, this is where the “many weak, few strong” pattern comes from: extinctions of most species have minor effects, while removal of those species that are strongly connected can destabilize the entire ecosystem, resulting in a cascade of extinctions. This instability essentially arises from “successful” interactions that form in the evolving network through, for example, collaborative consortia. Such interdependencies in collaborations can ultimately lead to instability, since disturbing any one species in the consortium can affect many others.

These features are among the self-organizing principles that reveal themselves in many natural and simulated networks. This suggests that the study of evolving networks can enable one to predict microbial ecosystem behavior, even without quantifying all the details of the interactions between species in a complex ecological network. When studying the complex communities of the human microbiome, where very little is known, this is a great advantage.

The application of theoretical network modeling to real ecological networks has thus far been focused primarily on attempts to capture observed features of the networks. One of the reasons for the rapid growth of network theory is the stunning regularity with which certain course-grained “topological” properties emerge in real ecological (and social and technological) networks. These properties, depending on global characteristics of the network such as the number of links, connectance, and so on, appear in such a wide variety of settings that it was natural to try to come up with simple models that would produce the same features. Thus, there appear both static and dynamic models that reproduce some of the topological properties of real networks [44, 45, 47, 48]. As one moves to more fine-grained properties (e.g., degree distribution) or seeks to develop predictive models, however, one must rely increasingly on dynamic models that carry more details about the system. Most studies of real ecological networks are restricted to food webs wherein all links between species are of the predator-prey type.

An example of how the models are applied to the real networks is in trying to understand the stability of an ecosystem to extinctions or other perturbations. Some models predict stability or instability based on the connectivity of the network. For example, the scale free property observed in many real food webs carries with it a prediction of stability under removal/extinction of weakly connected species but become highly unstable with avalanches of secondary extinctions when one of the few highly connected species is removed. There are limitations, however, to our current understanding since the stability analyses have been rather restricted and the models lack some details that could play essential roles.

## 6. SUMMARY

We have argued that mathematical networks provide a system-level approach to characterizing microbes and microbial interactions, which may improve descriptions of how consortia in the human microbiome are related to disease etiology, diagnosis, and treatment. Networks may capture specific biological information, such as how nutrients flow through the species in a microbial consortium. Ecological principles applied to such microbiome-specific networks are likely to constrain how the microbiome will respond to invasive species or to purportedly benign disturbances such as antibiotic treatment. Moreover, network structure sometimes suffices to indicate how a consortium is likely to have evolved or to identify keystone species, even when interaction strengths have not been quantified. This is particularly useful when detailed data on the constituents and species interactions in a consortium are unavailable. In short, for some human diseases such as bacterial vaginosis, it may be more useful to examine the forest, rather than the trees.
